# Supplementary material for: ROS-mediated apoptosis in colon cancer cells induced by sulfated polysaccharides from Gracilaria corticata
Source: PLoS One. 2026 May 18;21(5):e0344172. doi: 10.1371/journal.pone.0344172 (PMC13183202; doi:10.1371/journal.pone.0344172)
Supplement: Attachment — Submitted filename: Revision PONE.docx [file pone.0344172.s001.docx]

This manuscript entitled “Targeting colon cancer through apoptosis: the antioxidant and anticancer potential of sulfated polysaccharides from *Gracilaria corticate*”, explores the contribution of marine-derived bioactive compounds for potential pharmaceutical applications. The manuscript is publishable with some refinement as there are several structural, language, and scientific issues that need attention before publication. Here are my comments:

1. The title is overly descriptive. It could be modified to “ROS-mediated apoptosis in colon cancer cells induced by sulfated polysaccharides from *Gracilaria corticata*”, or a similar one.
2. Abstract: …rich source of these bioactive compounds…. Which bioactive compounds are indicated here? Please correct the sentence.
3. Be consistent in the use of abbreviations and units (such as SPs, CRC etc. and minutes to min, hours to h, ml or mL? microliters to μL and so on.), including the abstract.
4. Section 1, antioxidant effects are described multiple times in the 1^st^ and 2^nd^ paragraph, please trim the repetitive content. Elaborate the rationale for choosing G. corticata specifically (e.g., what makes its SPs unique?).
5. Headings# 3.2 and 3.4, red seaweed or red algae! please indicate only one terminology for G. corticata, just G. corticata is sufficient to mention.
6. Please check the reference#26 mentioned in 3.5.1., is it correct!
7. Please correct the headers (e.g., “A method for…”). MTT is related to 3.6.1 only and not to other subsections. Please correct the header for this section (3.6). The sentence… While cells in the other two groups … untreated HT-29 cells serving as control cells, is unclear and grammatically incorrect.
8. There are several issues in section 3.5.2. clearly distinguish between mM (molarity) and μg/mL (mass/volume) for ABTS solution. …to adsorb 1.1 at… please correct it, is it absorb? Adsorption??
9. Results- please stay consistent about units, mg or μg/mL when comparing the results.
10. Only extraction time is varied for molecular weight, despite the previous optimization involving three variables. The effect of power and solvent ratio is needed to be discussed. The mechanism of degradation under ultrasonic treatment is not discussed too.
11. Figures are lacking the visual clarity. Please upgrade.
12. While mentioning “selective anticancer activity”, more anticancer cell lines must be used to emphasize the selectivity.
13. There is inconsistency in Yield Values, discussion says 34.8%, while results section earlier claimed 27.8%.
14. The idea that sulfate or galactose content affects bioactivity is mentioned but not deeply explored. Provide more concrete evidences or reference studies that specifically correlate certain structural motifs with activity.
15. Concerning the extraction cost or feasibility, no practical comments are made on scalability or industrial applicability. Briefly mention challenges or advantages of ultrasound-assisted extraction in large-scale settings.
16. Italicize ‘in vivo’.
17. Conclusion- Add a sentence summarizing the unique contribution of SPs.
