# Supplementary material for: ROS-mediated apoptosis in colon cancer cells induced by sulfated polysaccharides from Gracilaria corticata
Source: PLoS One. 2026 May 18;21(5):e0344172. doi: 10.1371/journal.pone.0344172 (PMC13183202; doi:10.1371/journal.pone.0344172)
Supplement: Attachment — Submitted filename: Plos One Sep.docx [file pone.0344172.s002.docx]

**Review report**

The authors investigated the anti-MRSA potential of metabolites from *Solanum muricatum*. The authors successfully combine classical natural product chemistry—involving the isolation and structural elucidation of eight compounds—with bioassays for antimicrobial and anti-biofilm activity. This experimental work is complemented by an extensive in silico analysis, including protein-protein interaction (PPI) network analysis, molecular docking, and molecular dynamics (MD) simulations, to propose a mechanism of action for the most active compound. The integration of wet-lab and computational approaches is a significant strength of this study. The identification of kaempferol 3-gentiobioside (compound 1) as a potent agent against MRSA, along with its potential inhibitory action on the aminoglycoside phosphotransferase APH(3')-IIIa, is a noteworthy finding. While the manuscript is a valuable contribution to the field of natural product-based drug discovery, several major and minor points require clarification and revision before it can be considered for publication.

**Major comments**

**Geographical Framing:** The abstract and introduction prominently feature the rising incidence of MRSA in Ethiopia as the primary motivation for the study. However, the plant material was collected in Beni-Suef, Egypt (Methods, section 2.1). This creates a logical disconnect. The authors should revise the introduction to either provide a clear rationale for linking an Ethiopian public health problem to a plant collected in Egypt or, more appropriately, frame the study's motivation in the context of the global MRSA threat, using the situation in Ethiopia as one example among others.

**In-Silico Target (APH(3')-IIIa):** The manuscript abruptly transitions to the molecular docking analysis against 3',5"-aminoglycoside phosphotransferase type IIIa (APH(3')-IIIa). The rationale for selecting this specific enzyme as the primary target is not sufficiently justified. While it is a known resistance enzyme, the authors should explicitly state why it is the most relevant target for their study (see Antioxidant and antimicrobial properties of *Codium fragile* (Suringar) methanol extract: Insights from molecular docking analysis. (2024). *Algal research*. 103619. <https://doi.org/10.1016/j.algal.2024.103619>

- Is APH(3')-IIIa the predominant aminoglycoside resistance mechanism in the specific MRSA strain used (ATCC 43300)? A reference supporting this would be necessary.
- The PPI network analysis is extensive, but its role in identifying APH(3')-IIIa as the key target is unclear. The manuscript should clarify how the network analysis informed the choice of the docking target or if the two analyses were conducted in parallel.

**Anti-biofilm activity and proposed mechanism:** The study demonstrates that compounds 1, 2, and 3 have significant anti-biofilm activity. However, the entire in-silico investigation focuses on inhibiting APH (3')-IIIa, an enzyme related to antibiotic resistance, not directly to biofilm formation. The discussion fails to connect these two key findings. The authors should discuss whether the inhibition of APH(3')-IIIa is known to affect biofilm formation or if they hypothesize a separate, unexplored mechanism for the anti-biofilm effects. This is a critical gap in the narrative linking the experimental results to the mechanistic investigation.

**Synergistic activity results:** There is a direct contradiction between the data in Table 2 and the accompanying text.

- Line 325 states, "...while the combination of compound 1 with compound 3 showed additive activity."
- However, Table 2 reports a FICI of 0.351 for the combination of Comp. 1 and Comp. 3. According to the authors' own criteria (FICI ≤ 0.5 = synergistic), this result indicates synergy, not an additive effect. This discrepancy must be corrected in the text.

**Minor comments**

1. **Figure 2 :** The caption for Figure 2 is inconsistent. It states "B: compound B," but based on the results and logic, this should refer to compound 2. Please correct the caption to read "B: compound 2".
2. **Abstract:** The abstract is very dense with specific numerical data (e.g., MIC values, percentage ranges for biofilm reduction). While informative, it could be streamlined for better readability and broader impact by focusing more on the overall findings and their significance.
3. **Methods:** The manuscript states that compounds were identified based on spectral data and comparisons with literature. To ensure reproducibility and support the structural assignments, the authors should confirm that the raw spectral data (e.g., 1D and 2D NMR spectra for compounds 1-8), referenced as Figures S1-S14, will be provided in the Supporting Information.
4. **PPI Network Figures and Descriptions:** Figures 4 and 5, along with their descriptions, are very dense. While the analysis is thorough, the main text could be made more accessible by moving some of the detailed network descriptions to the supplementary materials and focusing the main discussion on the key pathways and hub proteins that are most relevant to the study's hypothesis.
5. **Typographical errors:** The manuscript would benefit from a thorough proofreading to correct minor grammatical errors and typos. For example:
   - Line 637: "afforded afforded" is a duplication.
   - Throughout the text, consistency in using symbols (e.g., µM vs. uM) should be checked.
6. **Conclusion:** The conclusion is largely a restatement of the results. The authors should expand this section to discuss the broader implications of their findings. What are the potential next steps (e.g., in vivo studies, lead optimization)? How do these flavonoid glycosides compare to other known natural product inhibitors of MRSA or resistance enzyme.
