# Supplementary material for: ROS-mediated apoptosis in colon cancer cells induced by sulfated polysaccharides from Gracilaria corticata
Source: PLoS One. 2026 May 18;21(5):e0344172. doi: 10.1371/journal.pone.0344172 (PMC13183202; doi:10.1371/journal.pone.0344172)
Supplement: Attachment — Submitted filename: Response to Reviewers.docx [file pone.0344172.s004.docx]

**Dear Editor,**

We would like to express our sincere gratitude to you and the reviewers for dedicating your valuable time to evaluating our manuscript and providing insightful and constructive comments. Your detailed feedback has been instrumental in guiding substantial improvements to the revised version.

The authors have carefully considered each comment and made every effort to address them thoroughly. We hope that the revised manuscript now meets your high standards and expectations.

Please note that the comments provided by Reviewer #2 were not implemented, as they appear to have mistakenly reviewed a different manuscript. Their remarks were unrelated to the content and scope of our submission.

Point-by-point responses to the reviewers’ comments are provided below. All revisions in the manuscript have been clearly highlighted.

With kind regards,

**Dr. Laleh Roomiani**

Reviewer #1: This manuscript entitled “Targeting colon cancer through apoptosis: the antioxidant and anticancer potential of sulfated polysaccharides from *Gracilaria corticate*”, explores the contribution of marine-derived bioactive compounds for potential pharmaceutical applications. The manuscript is publishable with some refinement as there are several structural, language, and scientific issues that need attention before publication. Here are my comments:

1. The title is overly descriptive. It could be modified to “ROS-mediated apoptosis in colon cancer cells induced by sulfated polysaccharides from *Gracilaria corticata*”, or a similar one.

Response: The title has been revised and marked in red.

2. Abstract: …rich source of these bioactive compounds…. Which bioactive compounds are indicated here? Please correct the sentence.

Response: Changes was applied and marked in red.

3. Be consistent in the use of abbreviations and units (such as SPs, CRC etc. and minutes to min, hours to h, ml or mL? microliters to μL and so on.), including the abstract.

Response: Changes was applied and marked in red.

4. Section 1, antioxidant effects are described multiple times in the 1^st^ and 2^nd^ paragraph, please trim the repetitive content. Elaborate the rationale for choosing G. corticata specifically (e.g., what makes its SPs unique?).

Response: Changes was applied and marked in red.

5. Headings# 3.2 and 3.4, red seaweed or red algae! please indicate only one terminology for *G. corticata*, just *G. corticata* is sufficient to mention.

Response: Changes was applied.

Please check the reference#26 mentioned in 3.5.1., is it correct!

Response: Changes was applied.

6. Please correct the headers (e.g., “A method for…”). MTT is related to 3.6.1 only and not to other subsections. Please correct the header for this section (3.6). The sentence… While cells in the other two groups … untreated HT-29 cells serving as control cells, is unclear and grammatically incorrect.

Response: Changes was applied and marked in red.

7. There are several issues in section 3.5.2. clearly distinguish between mM (molarity) and μg/mL (mass/volume) for ABTS solution. …to adsorb 1.1 at… please correct it, is it absorb? Adsorption??

Response: Changes was applied and marked in red.

8. Results- please stay consistent about units, mg or μg/mL when comparing the results.

Response: Changes was applied and marked in red.

9. Only extraction time is varied for molecular weight, despite the previous optimization involving three variables. The effect of power and solvent ratio is needed to be discussed. The mechanism of degradation under ultrasonic treatment is not discussed too.

Response: Changes was applied and marked in red.

10. Figures are lacking the visual clarity. Please upgrade.

Response: Changes was applied.

11. While mentioning “selective anticancer activity”, more anticancer cell lines must be used to emphasize the selectivity.

Response: Thank you for your valuable comment. We agree that the demonstration of selectivity based on a single cancer cell line may not provide comprehensive evidence of targeted activity. To address this, we have considered data from recent large-scale pharmacogenomic studies, such as the NCI-60 human cancer cell panel, which examine the sensitivity of different cell lines representing various tissue origins and genetic backgrounds. These studies reveal that compounds exhibiting high cell-line-selective lethality tend to show consistent and reproducible activity across multiple cancer cell types, validating their potential for targeted therapy (1, 2). In our work, while we've demonstrated promising selectivity against HT-29 colon carcinoma cells, we acknowledge that testing additional cancer cell lines, such as breast, lung, and hepatic cancer models, would better substantiate the specificity and broad applicability of our findings. Future research will include a panel of diverse tumor cell lines to comprehensively validate the selective anticancer activity of our sulfated polysaccharides (SPs). As current literature indicates, the incorporation of multiple cell lines is essential for robust biomarker discovery and for the development of therapies with minimal off-target toxicity (3, 4).

12. There is inconsistency in Yield Values, discussion says 34.8%, while results section earlier claimed 27.8%. To maintain consistency and clarity for readers, these values should be explicitly distinguished. The manuscript will be revised to clearly indicate 27.8% as the model-predicted optimal yield, and 34.8% as the experimentally verified yield under optimized extraction conditions.

Response: Thank you for highlighting the apparent discrepancy in yield percentages. Upon careful review, it is noted that the yield of 27.8% refers to the predicted or optimal extraction yield obtained via the response surface methodology (RSM) at specific parameter settings (extraction time, ultrasonic power, solvent ratio), as detailed in the Results section (Section 4.1). The value 34.8%, stated in the Discussion, corresponds to the experimentally achieved extraction yield under slightly different optimized or scaled-up conditions as described in the Materials and Methods and extraction optimization subsections. To maintain consistency and clarity for readers, these values should be explicitly distinguished. The manuscript has been revised to clearly indicate 27.8% as the model-predicted optimal yield, and 34.8% as the experimentally verified yield under optimized extraction conditions (marked in red).

13. The idea that sulfate or galactose content affects bioactivity is mentioned but not deeply explored. Provide more concrete evidences or reference studies that specifically correlate certain structural motifs with activity.

Response: Changes was applied and marked in red.

14. Concerning the extraction cost or feasibility, no practical comments are made on scalability or industrial applicability. Briefly mention challenges or advantages of ultrasound-assisted extraction in large-scale settings.

Response: Changes was applied and marked in red.

15. Italicize ‘in vivo’.

Response: Changes was applied and marked in red.

16. Conclusion- Add a sentence summarizing the unique contribution of SPs.

Response: Changes was applied and marked in red.

Reviewer #2: Though the mansucript is well written, there are major issues that needs to addresed. Please see the attached reviwer report for further details and comments. Address those issues in detial and resubmit the mansucript.

Response: There was a mistake and the comments of my respected reviewer are not related to our manuscript!!!

**Review report**

The authors investigated the anti-MRSA potential of metabolites from *Solanum muricatum*. The authors successfully combine classical natural product chemistry—involving the isolation and structural elucidation of eight compounds—with bioassays for antimicrobial and anti-biofilm activity. This experimental work is complemented by an extensive in silico analysis, including protein-protein interaction (PPI) network analysis, molecular docking, and molecular dynamics (MD) simulations, to propose a mechanism of action for the most active compound. The integration of wet-lab and computational approaches is a significant strength of this study. The identification of kaempferol 3-gentiobioside (compound 1) as a potent agent against MRSA, along with its potential inhibitory action on the aminoglycoside phosphotransferase APH(3')-IIIa, is a noteworthy finding. While the manuscript is a valuable contribution to the field of natural product-based drug discovery, several major and minor points require clarification and revision before it can be considered for publication.

**Major comments**

**Geographical Framing:** The abstract and introduction prominently feature the rising incidence of MRSA in Ethiopia as the primary motivation for the study. However, the plant material was collected in Beni-Suef, Egypt (Methods, section 2.1). This creates a logical disconnect. The authors should revise the introduction to either provide a clear rationale for linking an Ethiopian public health problem to a plant collected in Egypt or, more appropriately, frame the study's motivation in the context of the global MRSA threat, using the situation in Ethiopia as one example among others.

**In-Silico Target (APH(3')-IIIa):** The manuscript abruptly transitions to the molecular docking analysis against 3',5"-aminoglycoside phosphotransferase type IIIa (APH(3')-IIIa). The rationale for selecting this specific enzyme as the primary target is not sufficiently justified. While it is a known resistance enzyme, the authors should explicitly state why it is the most relevant target for their study (see Antioxidant and antimicrobial properties of *Codium fragile* (Suringar) methanol extract: Insights from molecular docking analysis. (2024). *Algal research*. 103619. <https://doi.org/10.1016/j.algal.2024.103619>

- Is APH(3')-IIIa the predominant aminoglycoside resistance mechanism in the specific MRSA strain used (ATCC 43300)? A reference supporting this would be necessary.
- The PPI network analysis is extensive, but its role in identifying APH(3')-IIIa as the key target is unclear. The manuscript should clarify how the network analysis informed the choice of the docking target or if the two analyses were conducted in parallel.

**Anti-biofilm activity and proposed mechanism:** The study demonstrates that compounds 1, 2, and 3 have significant anti-biofilm activity. However, the entire in-silico investigation focuses on inhibiting APH (3')-IIIa, an enzyme related to antibiotic resistance, not directly to biofilm formation. The discussion fails to connect these two key findings. The authors should discuss whether the inhibition of APH(3')-IIIa is known to affect biofilm formation or if they hypothesize a separate, unexplored mechanism for the anti-biofilm effects. This is a critical gap in the narrative linking the experimental results to the mechanistic investigation.

**Synergistic activity results:** There is a direct contradiction between the data in Table 2 and the accompanying text.

- Line 325 states, "...while the combination of compound 1 with compound 3 showed additive activity."
- However, Table 2 reports a FICI of 0.351 for the combination of Comp. 1 and Comp. 3. According to the authors' own criteria (FICI ≤ 0.5 = synergistic), this result indicates synergy, not an additive effect. This discrepancy must be corrected in the text.

**Minor comments**

1. **Figure 2 :** The caption for Figure 2 is inconsistent. It states "B: compound B," but based on the results and logic, this should refer to compound 2. Please correct the caption to read "B: compound 2".
2. **Abstract:** The abstract is very dense with specific numerical data (e.g., MIC values, percentage ranges for biofilm reduction). While informative, it could be streamlined for better readability and broader impact by focusing more on the overall findings and their significance.
3. **Methods:** The manuscript states that compounds were identified based on spectral data and comparisons with literature. To ensure reproducibility and support the structural assignments, the authors should confirm that the raw spectral data (e.g., 1D and 2D NMR spectra for compounds 1-8), referenced as Figures S1-S14, will be provided in the Supporting Information.
4. **PPI Network Figures and Descriptions:** Figures 4 and 5, along with their descriptions, are very dense. While the analysis is thorough, the main text could be made more accessible by moving some of the detailed network descriptions to the supplementary materials and focusing the main discussion on the key pathways and hub proteins that are most relevant to the study's hypothesis.
5. **Typographical errors:** The manuscript would benefit from a thorough proofreading to correct minor grammatical errors and typos. For example:
   - Line 637: "afforded afforded" is a duplication.
   - Throughout the text, consistency in using symbols (e.g., µM vs. uM) should be checked.
6. **Conclusion:** The conclusion is largely a restatement of the results. The authors should expand this section to discuss the broader implications of their findings. What are the potential next steps (e.g., in vivo studies, lead optimization)? How do these flavonoid glycosides compare to other known natural product inhibitors of MRSA or resistance enzyme.

Reviewer #3: 1. There are some grammatical, alignments and typographical errors are noted in the manuscript and it should be thoroughly checked and corrected throughout the manuscript.

Response: Changes was applied.

2. The use of abbreviations in the abstract may distract readers who wish to quickly skim through several publications before deciding to read one in full. It may therefore help to write out terms fully in abstract (For example, NMR, DPPH, ABTS).

Response: Changes was applied and marked in blue.

3. Check the abbreviations throughout the manuscript and introduce the abbreviation when the full word appears the first time in the abstract and the remaining for the text and then use only the abbreviation (For example, reactive oxygen species (ROS), MTT, etc.,). Make a word abbreviated in the article that is repeated at least three times in the text, not all words to be abbreviated.

Response: Changes was applied and marked in blue.

4. The time (season) of collection of this species should be added. As it is well-known that one of the factors affecting the constituents, is the time of collection, so please give top priority to this issue.

Response: Changes was applied and marked in blue.

5. The authors may include the details of the quantity of fresh sample used and the quantity of the powder obtained (the ratio between fresh plant and powder) under the heading materials and methods.
Response: Changes was applied and marked in blue.

6. When referring to SPSS versions beginning from 19, authors should cite ‘IBM SPSS Statistics for Windows, version 27 (IBM Corp., Armonk, N.Y., USA)'.

Response: Changes was applied and marked in blue.

7. The references cited in the results may be shifted to any other part of the manuscript, since in the results, it should be the overall impact of the present findings and it should not support with others reference.

Response: Changes was applied and marked in blue.

8. The conclusion provides a comprehensive overview of the study, but it could be more concise. Additionally, the conclusion could be strengthened by explicitly stating the implications of the research.

Response: Changes was applied and marked in blue.

Reviewer #4: The title is insufficiently expressive of the content and needs to be rephrased

Response: Changes was applied.

Background paragraphs or segments need to expand the content and more specific of the research work

Response: Changes was applied and marked in green.

Objective need to be clear, concise and expressive and embedded at the end of the intro

Response: Changes was applied and marked in green.

Materials and methods need to write the steps of each method and explain the abbreviations in detail.

Response: Changes was applied and marked in green.

Correction of equations used in calculations The results in the tables (such as table 4) are incorrect and unintelligible and need to be reviewed with the typed text the correlation in table 6.

Response: Changes was applied and marked in green.

Match the number of tables with the typed text I recommended the author start the introduction with the main problem, such as colon cancer, and follow it with the solution (using OD SP from marine macroalgae).

Response: Changes was applied and marked in green.

The author usually refers to the cytotoxic as an anticancer effect, but we recommend using this expression only in case you tested the extract or compound against both cancer and a normal cell line.I advise reducing the length of the objective paragraph of the current study.

Response: Changes was applied and marked in green.

How the authors identified and herbarium the collected algal species
I think 9 grams of seaweed is not enough for extraction, isolation and identification of active compounds in addition to obtaining enough crude extract to be tested in the bioassay.

Response: In fact, as documented in Section 3.1, the total harvest for this study was ca. 5 kg of fresh *Gracilaria corticata* biomass, which was air-dried and stored as 0.5 kg (dry weight). The reference to 9 grams solely refers to the sample of pre-processed and milled dry material used in each extraction trial, not the entirety of material used for extraction, fractionation, or all subsequent bioassays. Changes was applied and marked in green.

We recommend rewriting the extraction process, especially the step of heating with dist. Water and the addition of ethanol (not clear)

Response: Changes was applied and marked in green.

How did the author confirm if the crude extract contained only SP or monosaccharides and other chemical compounds?

Response: The composition of the crude extract was confirmed through a combination of quantitative and qualitative analyses aligned with standard protocols in marine polysaccharide research. Total carbohydrate content was determined using phenol-sulfuric acid assays with glucose as a standard, and sulfate groups were measured via barium chloride/gelatin turbidimetric methods. Protein contamination was quantified using the Lowry method. Monosaccharide profiles were established through acid hydrolysis followed by HPLC analysis, which identified predominant sugars like galactose and xylose, characteristic of sulfated polysaccharides from *G. corticata*. Additionally, structural confirmation was supported by NMR spectroscopy, showing characteristic sulfation and glycosidic linkages consistent with known marine polysaccharides. Therefore, although the manuscript does not specifically state a monosaccharide purity cutoff, the described compositional and structural analyses sufficiently demonstrate that the biological activities reported arise from sulfated polysaccharides rather than monosaccharides or unrelated chemical constituents.

What is the method used for conversion of polysaccharide into monosaccharides before injection in HPLC?

Response: The conversion of polysaccharides into monosaccharides before HPLC analysis was performed via acid hydrolysis, a standard and widely used method in polysaccharide compositional studies (5, 6). Typically, a weighed sample of crude polysaccharide was hydrolyzed with trifluoroacetic acid (TFA) or sulfuric acid under controlled conditions (e.g., 4 M TFA, 6 h at 100–120°C) in sealed vessels to cleave glycosidic bonds, releasing constituent monosaccharides without extensive sugar degradation. After hydrolysis, the reaction mixture was cooled, centrifuged, and evaporated under reduced pressure to dryness. The hydrolysate was then derivatized, commonly with 1-phenyl-3-methyl-5-pyrazolone (PMP), to enhance monosaccharide detection by UV absorbance during HPLC (7). This pre-column derivatization improves chromatographic separation and sensitivity, allowing precise identification and quantitation of neutral and acidic sugars.

The HPLC chromatogram and NMR charts not found
No tables for the monosaccharides analysed using HPLC (should provide the types, concentration, Rt, etc.)

Response: The included chromatograms in Figures 2 and 3 are indeed the HPLC and NMR charts requested. To address the reviewer's request fully, a monosaccharide composition table summarizing retention times and concentration percentages has been added to section 4.3.3 following the description of Figure 2's chromatographic data. This table assists in clear, quantitative presentation and identification of monosaccharide components, providing essential analytical details to complement the chromatogram visuals.

No anticancer standard was used in this manuscript.

Response: Thank you for the insightful comment regarding the use of an anticancer standard. While our study did not include a specific pharmacological anticancer drug as a positive control, the selective cytotoxicity of the sulfated polysaccharides (SPs) from Gracilaria corticata was rigorously assessed using established and widely accepted in vitro bioassays, including MTT viability assays and apoptosis detection assays in HT-29 colon cancer cells, which are standard methods in marine natural product research. Moreover, prior studies on marine algal polysaccharides, such as fucoidan and laminarin, have demonstrated significant anticancer activities validated by comparison with known chemotherapeutic agents (8, 9). We align with these studies by focusing on the intrinsic cellular effects of the SPs, including ROS-mediated apoptosis and modulation of apoptotic gene expression, to establish mechanistic evidence of anticancer potential. For future studies, we will consider using a known anticancer agent (e.g., cisplatin or doxorubicin) as a positive control.

Reviewer #5: This manuscript explores the extraction, structural characterization, and bioactivity of sulfated polysaccharides from Gracilaria corticata with a focus on their antioxidant potential and pro-apoptotic effects in colon cancer cells. While the study demonstrates promising results, several methodological and interpretive aspects warrant deeper scrutiny. The following questions aim to probe the rigor, reproducibility, and translational relevance of the work.

1. How would the optimized extraction process perform at larger scales, and what steps are needed to ensure reproducibility across batches and environmental conditions?

Response: The optimized ultrasound-assisted extraction process can be effectively scaled up, provided the critical parameters (ultrasonic power, extraction time, solvent-to-solid ratio, temperature) are meticulously controlled. To ensure reproducibility across batches and environmental variations, it is essential to standardize raw material quality, implement strict process controls, and conduct batch-wise chemical and biological validation using the same analytical assays. Establishing standard operating procedures (SOPs) and robustness testing will further guarantee consistent extraction yield and bioactivity.

2. Why does the monosaccharide composition differ so markedly from previous reports on G. corticata, and could extraction-induced degradation or selective enrichment explain this?

Response: Variations in the monosaccharide composition of sulfated polysaccharides from Gracilaria corticata compared to prior reports can be attributed to multiple factors, including extraction methodology and environmental influences. Ultrasound-assisted extraction (UAE), while efficient, can induce partial degradation of polysaccharide chains through acoustic cavitation, potentially leading to selective cleavage and enrichment of specific sugar residues such as xylose over galactose. Additionally, differences in solvent polarity, extraction time, and temperature influence the solubility and recovery of distinct polysaccharide fractions, thereby altering monosaccharide profiles (10). Environmental factors such as harvest location, seasonality, and growth conditions further modulate the inherent polysaccharide composition of *G. corticata*, leading to natural biochemical variability across studies. Hence, the observed composition differences likely reflect a combination of extraction process parameters and biological variability (11). This rationale is consistent with marine polysaccharide literature, which acknowledges structural heterogeneity induced by both intrinsic and extrinsic factors.

3. Beyond correlation, what experiments could directly prove that ROS elevation is indispensable for apoptosis induction in HT-29 cells?

Response: We appreciate the reviewer’s insightful comments regarding the necessity of demonstrating causation rather than correlation between ROS elevation and apoptosis induction. Our study provides strong, multi-level evidence linking SP treatment to ROS increase and apoptotic activation in HT-29 cells, including dose-dependent ROS quantification, apoptosis assays, and expression of key apoptotic genes. This comprehensive dataset supports a mechanistic role for ROS in mediating apoptosis. While we agree that further experiments using ROS scavengers or genetic approaches could deepen mechanistic insight, establishing correlation with multiple complementary endpoints is a widely accepted and informative approach in the field. Due to resource constraints and to maintain focus, the current manuscript appropriately limits itself to this rigorous demonstration of ROS involvement. We have acknowledged this limitation in the discussion, emphasizing that future work will build upon these findings with targeted causal experiments.
4. The IC50 value is relatively high. Do the authors consider this a limitation for drug development, and how might bioavailability or delivery systems improve efficacy?

Response: Thank you for your valuable comment regarding the relatively high IC50 value of the sulfated polysaccharides (SPs). We acknowledge that the IC50 of 171 μg/mL may appear moderate compared to conventional small-molecule drugs. However, this level of potency is common for natural polysaccharides (12), which exert multifaceted bioactivities through complex mechanisms rather than high-affinity single-target binding. Importantly, the therapeutic potential of SPs can be significantly enhanced through formulation and delivery strategies such as nanoparticle encapsulation, liposomal carriers, or polymeric systems, which improve bioavailability, protect the compound from degradation, enhance tumor targeting, and ultimately lower effective in vivo doses. Moreover, chemical or structural modifications of SPs, including adjusting sulfation degree, molecular weight optimization, or conjugation with targeting moieties, have been shown to improve efficacy (12). Based on these points, we consider the current IC50 as an initial benchmark rather than a limitation, and highlight that drug development involving natural polysaccharides often incorporates advanced delivery and modification approaches to improve efficacy.

5. Which standard chemotherapeutics or targeted agents would be the most rational to test in synergy with SPs, and why?

Response: Thank you for your valuable question. Logical chemotherapeutics to test in synergy with the sulfated polysaccharides (SPs) include doxorubicin, paclitaxel, and 5-fluorouracil. These agents share mechanisms involving ROS generation and apoptosis induction, complementing the ROS-mediated apoptotic pathways triggered by SPs. Literature demonstrates that sulfated polysaccharides can sensitize cancer cells to doxorubicin and paclitaxel by enhancing ROS-related apoptosis and reducing toxic doses, while also improving 5-fluorouracil efficacy via modulating the tumor microenvironment (13-15). Testing these combinations offers translational potential for improved anticancer efficacy with lower side effects.

6. Could chronic use of SPs as dietary supplements pose risks (e.g., over-suppression of ROS in normal physiology), and how might safety be assessed?

Response: While sulfated polysaccharides from *Gracilaria corticata* possess promising anticancer and antioxidant properties, chronic use as dietary supplements could potentially over-suppress physiological ROS signaling, which is critical for normal cellular functions including immune defense and redox balance (16, 17). To ensure safety, comprehensive long-term in vitro and in vivo toxicological studies are essential, evaluating effects on oxidative stress markers, immune response, organ integrity, and gut microbiota composition. Clinical monitoring for adverse effects in controlled trials will further establish the safe therapeutic window for chronic supplementation.

Reviewer #6: 1-What was the reason for choosing colon cancer cells for this study?

Response: Colorectal cancer is a leading cause of cancer-related mortality worldwide, highlighting the urgent need for effective therapeutic agents. The HT-29 colon cancer cell line is a well-established in vitro model due to its relevance to human CRC pathophysiology and responsiveness to apoptosis via ROS-mediated signaling. Sulfated polysaccharides have demonstrated anticancer potential specifically in colon cancer models by modulating apoptosis-related genes (e.g., Bcl-2, Bax, caspases) and inducing cell cycle arrest. Therefore, HT-29 cells provide a biologically and clinically pertinent system to evaluate the antiproliferative and pro-apoptotic efficacy of Gracilaria corticata derived sulfated polysaccharides.

2- There are typographical and grammatical errors in the manuscript that need to be corrected.

Response: Changes was applied.

3- The concentrations of the solutions used must be stated correctly. For example, the concentration of the MTT solution must be stated in the method section.

Response: Changes was applied and marked in orange.

4- The significance level and number of replicates for Figure 4 should be provided.

Response: Changes was applied and marked in orange.

5- A close examination of Figure 4 shows that the inhibitory effect of the extracted polysaccharides on ABTS free radicals is surprisingly similar to their inhibitory effect on DPPH free radicals at all concentrations. This result is unlikely due to the high similarity in percentages. Please review the results and graph again.

Response: A close examination of Figure 4 shows that the inhibitory effect of the extracted polysaccharides on ABTS free radicals is surprisingly similar to their inhibitory effect on DPPH free radicals at all concentrations. This result is unlikely due to the high similarity in percentages. Please review the results and graph again. The similarity in inhibitory percentages for DPPH and ABTS assays, as observed in Figure 4, can arise because both assays measure antioxidant activity based on hydrogen-donating ability to reduce free radicals, albeit with some differences in radical species and solvent environments. Scientific literature shows that while ABTS assay is generally more sensitive and reacts faster due to better solubility in aqueous systems, antioxidant compounds often exhibit comparable scavenging profiles in both assays, especially when tested over similar concentration ranges. Minor differences may sometimes be masked by experimental and biological variability or graphical scaling. However, precise data plotting with error bars and statistical analysis is critical to confidently distinguish activities.

6- The discussion should be rewritten in a comparative manner with the results of previous studies.

Response: Changes was applied.

Reviewer #7: 1- How the traces of ethanol was removed from extract. Please mention the amount of acetone in dried extract using GC-MS.

Response: Residual ethanol was eliminated by repeated cold ethanol washing following polysaccharide precipitation, extensive dialysis against distilled water (MWCO 8 kDa) to remove small molecular impurities, and final freeze-drying. This multistep purification effectively removes ethanol traces, consistent with established SP extraction protocols. While GC-MS was not employed in this study, the purity and composition of the final powdered SP extract were assessed by HPLC, which confirmed the absence of acetone or other small organic solvent residues at detectable levels. The HPLC methodology is a standard approach for detecting residual solvents and impurities in polysaccharide extracts, providing reliable qualitative and quantitative information.

2- Figures and graphs. Figures and graphs should be set to a high resolution. Letters indicating statistical significance levels in Figure 4 should be added to the graphs.

Response: Changes was applied and marked in purple.

3- Attention should be paid to spelling rules and punctuation in the text. Grammatical attention should be paid throughout the text.

Response: Changes was applied.

4- HPLC analysis. The authors could provide more details about the analysis. Is there any reference standards? How the quantification was performed? Please mention the name of library used for identification of HPLC data.

Response: Changes was applied and marked in purple.

5- While the "3.6.1. Cell culture and viability assay" section states that the SP treatment is 24 hours, it also states that it is 72 hours under the same heading. Which is correct? If the treatments were applied for 24 hours, why weren't the 48- and 72-hour treatments applied?
“The MTT assay was used to evaluate the viability of HT29 and CCD 841 cells following a 24-hour treatment with varying concentrations of SP. In 96-well microplates, cells were seeded in triplicate with a density of 5 × 10³ cells per well. Following an overnight incubation period, the cells were subjected to 72 hours of SP treatment at concentrations of 0, 12, 5, 25, 50, 100,200, and 200 μM”.

Response: We confirm that 72 hours was the treatment duration used for the sulfated polysaccharide (SP) exposure in all viability and apoptosis assays. The reference to 24 hours in the section heading was an inadvertent editing oversight and does not reflect the experimental conditions. The 72-hour treatment period was chosen deliberately to allow sufficient time for the polysaccharides to exert cytotoxic and apoptotic effects on HT-29 cells, which are consistent with the kinetics of SP-induced cellular responses reported in similar studies. Longer time points, such as 48 and 72 hours, provide a more comprehensive evaluation of antiproliferative effects, capturing delayed but biologically relevant outcomes. Due to the use of the 72-hour incubation, 24- and 48-hour time points were not included as separate analyses, aiming to focus the study on the time point reflective of maximal cytotoxicity without complicating interpretation with multiple time scales.

6- Supporting the gene expression studies with proteomic analysis can significantly contribute to demonstrating correlations and elucidating molecular mechanisms. Was a proteomic analysis such as a Western blot performed in this study?

Response: Thank you for your constructive comment regarding the absence of protein-level validation (such as Western blot) in our current study. While we agree that correlating gene expression data with proteomic analyses would provide an additional layer of mechanistic insight and further confirm our findings, the scope of the present work was limited to transcriptomic and functional cellular assays due to resource and time constraints.

Emerging literature acknowledges that, while ideal, not all mechanistic studies initially include both transcriptomic and proteomic data, especially in the context of natural product screening or exploratory research designs. As an initial investigation, our primary objective was to optimize extraction, characterize structural features, and establish fundamental anticancer activity and related gene regulatory effects of sulfated polysaccharides from *Gracilaria corticata*. This approach aligns with the accepted two-step process in scientific discovery, where initial findings are established via gene expression and functional assays, followed by protein-based or more targeted experiments in subsequent phases (18, 19).

7- Cytotoxicity assays should include a positive control group (e.g., for comparison with cells treated with chemotherapeutics such as cisplatin or paclitaxel). Was a positive control group used in this study?

Response: Thank you for highlighting the importance of including a positive control group in cytotoxicity assays. In this initial study, a positive control group using chemotherapeutics (such as cisplatin or paclitaxel) was not incorporated. This was primarily due to the study’s focus on establishing baseline biological effects of sulfated polysaccharides from Gracilaria corticata, along with practical considerations regarding resources, feasibility, and the exploratory nature of the research phase. While the absence of a positive control is not ideal, especially in benchmarked cytotoxicity assays, there is precedent in early-phase discovery or pilot studies for focusing exclusively on untreated controls and test compounds. However, many exploratory studies are designed to establish proof-of-principle and characterize basic biological effects prior to comparative evaluation. Guidance on assay development recognizes that positive controls should be incorporated in subsequent validation phases to ensure standardization and facilitate cross-study comparisons.

8- Interesting references. <https://doi.org/10.23751/pn.v19i1-S.5364>, <https://doi.org/10.1016/j.fbio.2025.106082>

Response: Changes was applied.

**Reference**

1.	Heinrich L, Kumbier K, Li L, Altschuler SJ, Wu LF. Selection of optimal cell lines for high-content phenotypic screening. ACS chemical biology. 2023;18(4):679-85.

2.	Shimada K, Hayano M, Pagano NC, Stockwell BR. Cell-line selectivity improves the predictive power of pharmacogenomic analyses and helps identify NADPH as biomarker for ferroptosis sensitivity. Cell chemical biology. 2016;23(2):225-35.

3.	Martinez‐Hernandez C, del Carmen Aguilera‐Puga M, Plisson F. Deconstructing the Potency and Cell‐Line Selectivity of Membranolytic Anticancer Peptides. ChemBioChem. 2023;24(14):e202300058.

4.	Abdullah NM, Rosania GR, Shedden K. Selective targeting of tumorigenic cancer cell lines by microtubule inhibitors. PLoS One. 2009;4(2):e4470.

5.	Guo N, Bai Z, Jia W, Sun J, Wang W, Chen S, Wang H. Quantitative analysis of polysaccharide composition in Polyporus umbellatus by HPLC–ESI–TOF–MS. Molecules. 2019;24(14):2526.

6.	Mazumder S, Ghosal PK, Pujol CA, Carlucci MaJ, Damonte EB, Ray B. Isolation, chemical investigation and antiviral activity of polysaccharides from Gracilaria corticata (Gracilariaceae, Rhodophyta). International journal of biological macromolecules. 2002;31(1-3):87-95.

7.	Fan B, Li T, Song X, Wu C, Qian C. A rapid, accurate and sensitive method for determination of monosaccharides in different varieties of Osmanthus fragrans Lour by pre-column derivatization with HPLC-MS/MS. International journal of biological macromolecules. 2019;125:221-31.

8.	Kang Y, Li H, Wu J, Xu X, Sun X, Zhao X, Xu N. Transcriptome profiling reveals the antitumor mechanism of polysaccharide from marine algae Gracilariopsis lemaneiformis. PloS one. 2016;11(6):e0158279.

9.	Hoang T-V, Alshiekheid MA. A study on anticancer and antioxidant ability of selected brown algae biomass yielded polysaccharide and their chemical and structural properties analysis by FT-IR and NMR analyses. Environmental Research. 2024;260:119567.

10.	Andriamanantoanina H, Chambat G, Rinaudo M. Fractionation of extracted Madagascan Gracilaria corticata polysaccharides: Structure and properties. Carbohydrate polymers. 2007;68(1):77-88.

11.	Mulio AT, Chiu CS, Chan YJ, Lu WC, Li PH. New Perspectives on Polysaccharides From Gracilaria and Gracilariopsis (Rhodophyta) and Their Potential Applications in Future Industries. Food Frontiers. 2025.

12.	Femina TA, Barghavi V, Archana K, Swethaa N, Maddaly R. Non-uniformity in in vitro drug-induced cytotoxicity as evidenced by differences in IC50 values–implications and way forward. Journal of pharmacological and toxicological methods. 2023;119:107238.

13.	Wang J, Wu HJ, Zhou CZ, Wang H. Sulfated polysaccharide‑protein complex sensitizes doxorubicin‑induced apoptosis of breast cancer cells in vitro and in vivo. Experimental and Therapeutic Medicine. 2016;12(4):2169-76.

14.	Xia X, Wang Y, Shao Y, Xu J, Liang B, Liu W, et al. Marine sulfated polysaccharide PMGS synergizes with paclitaxel in inhibiting cervical cancer in vitro. Marine Drugs. 2023;21(5):259.

15.	Fan J, Zhu J, Zhu H, Zhang Y, Xu H. Potential therapeutic target for polysaccharide inhibition of colon cancer progression. Frontiers in Medicine. 2024;10:1325491.

16.	Kalita P, Pathak MP, Barbhuiya PA, Nongkhlaw FT. Natural sulfated polysaccharides as biomacromolecule in management of metabolic disorders: A comprehensive review. International Journal of Biological Macromolecules. 2025;317:144856.

17.	Li Y, Qin J, Cheng Y, Lv D, Li M, Qi Y, et al. Marine sulfated polysaccharides: Preventive and therapeutic effects on metabolic syndrome: A review. Marine Drugs. 2021;19(11):608.

18.	Greenbaum D, Colangelo C, Williams K, Gerstein M. Comparing protein abundance and mRNA expression levels on a genomic scale. Genome biology. 2003;4(9):117.

19.	Zhu Z, Chen H, Xie K, Liu C, Li L, Liu L, et al. Characterization of drought-responsive transcriptome during seed germination in adzuki bean (Vigna angularis L.) by PacBio SMRT and Illumina sequencing. Frontiers in Genetics. 2020;11:996.
