# Supplementary material for: ROS-mediated apoptosis in colon cancer cells induced by sulfated polysaccharides from Gracilaria corticata
Source: PLoS One. 2026 May 18;21(5):e0344172. doi: 10.1371/journal.pone.0344172 (PMC13183202; doi:10.1371/journal.pone.0344172)
Supplement: Attachment — Submitted filename: Reviewer response (2).docx [file pone.0344172.s005.docx]

**Dear Editor,**

We would like to express our sincere gratitude to you and the reviewers for dedicating your valuable time to evaluating our manuscript and providing insightful and constructive comments. Your detailed feedback has been instrumental in guiding substantial improvements to the revised version.

The authors have carefully considered each comment and made every effort to address them thoroughly. We hope that the revised manuscript now meets your high standards and expectations.

Please note that the comments provided by Reviewer #2 were not implemented, as they appear to have mistakenly reviewed a different manuscript. Their remarks were unrelated to the content and scope of our submission.

Point-by-point responses to the reviewers’ comments are provided below. All revisions in the manuscript have been clearly highlighted.

With kind regards,

**Dr. Laleh Roomiani**

Reviewer #1: Although the authors have addressed most of the previous comments and revised other sections accordingly, the quality of several figures (e.g., Figures 2, 3, and 5C) remains insufficient. The low resolution and unclear labeling hinder proper data interpretation. High-resolution figures with clearly legible annotations are still required.

Response: Change was applied.

Reviewer #3: 1. This suggestion is not carried out properly and it should be rectified. The use of abbreviations in the abstract may distract readers who wish to quickly skim through several publications before deciding to read one in full. It may therefore help to write out terms fully in abstract (For example, NMR, DPPH, ABTS, but given expansion in next para for DPPH and ABTS). The same may be considered in the title also.

Response: Change was applied and marked in red.

2. This suggestion is not carried out properly and it should be rectified (Check the abbreviations throughout the manuscript and introduce the abbreviation when the full word appears first time in the abstract and the remaining for the text and then use only the abbreviation). For example, reactive oxygen species (ROS), MTT, and these types of corrections need to be checked all other abbreviations used in the manuscript.

Response: We sincerely thank the reviewer for highlighting this important issue regarding the consistency and correct introduction of abbreviations throughout the manuscript. We fully agree that a proper definition of abbreviations at their first occurrence, particularly in the abstract, is essential for clarity, readability, and adherence to scientific writing standards. Accordingly, the entire manuscript has been carefully re-examined to ensure that each abbreviation is introduced only once at its first appearance with the full term, after which the abbreviated form is used consistently.

3. This suggestion is not carried out properly and it should be rectified. When referring to SPSS versions beginning from 19, authors should cite ‘IBM SPSS Statistics for Windows, version 27 (IBM Corp., Armonk, N.Y., USA)'.

Response: Change was applied and marked in red.

4. This suggestion is not carried out properly and it should be rectified. The references cited in the results may be shifted to any other part of the manuscript, since in the results, it should be the overall impact of the present findings and it should not support with others reference.

Response: Change was applied and marked in red.

5. The conclusion seems in general and it should be cocise. All conclusions must be convincing statements on what was found to be novel impact based on the strong support of the data/results/discussion.

Response: Change was applied and marked in red.
